# Understanding the Natural Language of DNA using Encoder-Decoder Foundation Models with Byte-level Precision

**Published:** 2024-02-14

**Authors:** Aditya Malusare, Harish Kothandaraman, Dipesh Tamboli, Nadia A. Lanman, Vaneet Aggarwal

**Affiliations:** Purdue University, West Lafayette IN, USA 47907

**Keywords:** DNA, Transformers, Attention, Mutations, Gene Annotation

## Abstract

This paper presents the Ensemble Nucleotide Byte-level Encoder-Decoder (ENBED) foundation model, analyzing DNA sequences at byte-level precision with an encoder-decoder Transformer architecture. ENBED uses a sub-quadratic implementation of attention to develop an efficient model capable of sequence-to-sequence transformations, generalizing previous genomic models with encoder-only or decoder-only architectures. We use Masked Language Modeling to pre-train the foundation model using reference genome sequences and apply it in the following downstream tasks: (1) identification of enhancers, promotors and splice sites, (2) recognition of sequences containing base call mismatches and insertion/deletion errors, an advantage over tokenization schemes involving multiple base pairs, which lose the ability to analyze with byte-level precision, (3) identification of biological function annotations of genomic sequences, and (4) generating mutations of the Influenza virus using the encoder-decoder architecture and validating them against real-world observations. In each of these tasks, we demonstrate significant improvement as compared to the existing state-of-the-art results.

## Introduction

The rise of foundation models in recent years has led to tremendous developments in understanding natural languages [26]. Although they were originally developed to process and generate written text, these models have transcended their initial purpose due to their generalizable nature and wide applicability. Foundation models have shown great potential in the field of bioinformatics [36], since their capacity to be trained on vast amounts of unlabeled data and their adaptability enable them to achieve state-of-the-art performance in a variety of tasks.

Early applications of foundation models in bioinformatics can be seen in analyzing protein sequences [9, 27], which were then trained on diverse applications like calculation of protein structure, prediction of mutation effects and the understanding of phylogenetic structure [20, 10, 24]. These models have since evolved beyond proteins into DNA and RNA analysis, and have demonstrated the ability to surpass previous benchmarks in identifying regulatory elements, predicting chromatin profiles, analyzing evolution from genomic sequence data and predicting the impacts of mutations in DNA [19, 7, 23, 39, 34]. The ability to visualize and interpret the internal model structure [32] and to derive key insights of the underlying biological processes [37] demonstrate the unique advantages offered by foundation models in the field of bioinformatics.

### Limitations of previous work

#### Architecture.

Prior work on Transformer-based models for DNA sequence analysis exists in two forms: (i) Encoder-only models [19, 11, 37, 7] that focus on classification and regression-based downstream tasks and (ii) Decoder-only models [23, 35] that are capable of classification, regression as well as generative tasks that involve design and synthesis.

A combination of encoder and decoder blocks enables the model to perform sequence-to-sequence transformations. One of the fundamental processes undergone by DNA is its transcription into an RNA sequence and subsequent translation into protein sequences, the building blocks of all living organisms. Understanding sequence-to-sequence processes like these is crucial to advancing our knowledge of genetics, and developing an encoder-decoder model is an important step in this direction. Although decoder-only models are capable of sequence-to-sequence transformations, they have no independent means of creating representations of the input sequence, and both input and target tokens are processed in an equivalent fashion. Previous work has shown that a multitask finetuned encoder-decoder Large Language Model (LLM) outperforms decoder-only models on zero-shot generalization, despite being an order of magnitude smaller [28]. Since a decoder-only architecture will have a unidirectional framework that attends to the source and target sequence simultaneously, as the length of the target sequence grows, the extent to which the model attends to the source will decrease leading to reduced performance in downstream tasks [13]. Our work demonstrates how the cross-attention layers in the decoder leverage the information in the embeddings generated by the encoder, leading to improved performance in training tasks.

#### Tokenization.

Biological sequences like DNA are encoded using a vocabulary of four symbols (A, C, T, G) representing nucleic acids. These sequences are converted into a Transformer-compatible format by a tokenizer, which generates a list of tokens for any given input. Since these models were initially developed for applications in natural languages, the most prevalent forms of tokenization are sentence-piece or word-piece, where the language vocabulary is built using natural ideas like words or syllables. In the absence of typical indicators of linguistic order in DNA, like spaces and punctuation, these tokenization schemes use statistical techniques to determine the ‘words’ that make up the vocabulary of the input sequences. A few examples of previously used tokenizers are: k-mer [19], SentencePiece [7], and byte-pair encoding (BPE) [11] tokenization. While such techniques identify optimal encoding methods by constructing tokens having multiple base pairs, they are vulnerable to any type of noise present in the sequence. A single variation in a base pair will result in the fragment being mapped to a completely different word in the vocabulary, resulting in an outsized impact from a small perturbation [8]. We use a simplified tokenization scheme where each character corresponds to a single token, resulting in a longer average tokenized length, but more resiliance to the variations mentioned above.

### Our contributions

In this paper, we develop the Ensemble Nucleotide Byte-level Encoder-Decoder (**ENBED**) Transformer, a foundation model that analyzes nucleotide sequences with Transformers using byte-level tokenization and an encoder-decoder model. This implementation bridges the gap between existing models that are either encoder-only or decoder-only implementations and presents the possibility of sequence-to-sequence analysis tasks. Using sliding-window and global attention we obtain a sub-quadratic implementation of attention, and demonstrate the performance improvements over dense attention. The foundation model is pre-trained using an ensemble of high-quality reference genomes from NCBI RefSeq, including the telomere-to-telomere assemblies of Human and Maize DNA, data from the 1000 Genomes Project and a mix of widely studied organisms like *E. coli*, *D. melanogaster*, *M. musculus* and *P. vivax* (Sec 6). This process is implemented by giving the model a self-supervised goal of internalizing the structure of the language of nucleotide sequences.

ENBED is built using a byte-level tokenizer. In order to avoid the issues created by single nucleotide variants and their downstream impacts, we side-step the problem of determining the tokenization scheme entirely by working with single nucleotides as tokens. This leads to increased computational costs, but grants resilience to the types of variations and noise commonly encountered in DNA sequences. In order to offset the impact of increased computations, we implement sub-quadratic attention layers in order to scale up the model efficiently.

#### Evaluation of performance on genomic benchmark datasets.

We evaluate the performance of the ENBED foundation model on sequence-level classification tasks and compare it’s accuracy against contemporary foundation models. We show that ENBED outperforms the state-of-the-art in 22 of the 25 benchmarks devised by the authors of the Nucleotide Transformer [7] and Genomic Benchmarks [15] datasets. These benchmarks consist of tasks like identifying enhancers, promotors, splice sites and histone marks in multi-species data comprising of genomic sequences from human, mouse, yeast, fruit fly and worm DNA.

#### Identifying sequencing noise.

Long-read sequencing using Nanopores is used to study telomeres, which are protective caps found at chromosomal ends and have long repetitive elements. It has been found that telomeres in many organisms are frequently miscalled [30], referring to errors in the process that translates electrical signals into the alphabet of DNA. We illustrate how ENBED can focus on fragments that look incorrect or out of place, demonstrating the model’s ability of distinguishing between noisy and accurate data. In a synthetic dataset constructed using noise distributions found in real-world raw sequence data, we demonstrate that our model can identify sequences containing noise with an accuracy of 97.1%, leveraging the information internalized by bring pretrained on the telomere-to-telomere reference sequences.

#### Biological function annotations.

Mapping the complete human genome was a significant milestone in modern biology, and it has produced a new set of challenges in identifying the functions and interactions of different parts of the genome. We fine-tune our model to solve a version of this problem by identifying the biological functions of genomic sequences among the nine most common functional classes using a fine-tuned model, achieving an $F_{1}$ score of 74.1.

#### Studying mutations as a sequence-to-sequence process.

Exploring mutations is essential as it sheds light on the mechanisms driving genetic diversity which enhance the overall resilience of living organisms in a changing environment. The encoder-decoder architecture confers the ability to rapidly iterate mutagenization of genomic segments. We study mutations in the Influenza virus, using the NCBI Influenza Virus Resource. By constructing a dataset with a phylogenetic tree, we obtain parent-child pairs of mutated sequences and show the effectiveness of our encoder-decoder architecture in analyzing and predicting these mutations.

## Methods

### Encoder-Decoder Model Architecture

**Fig. 1.**
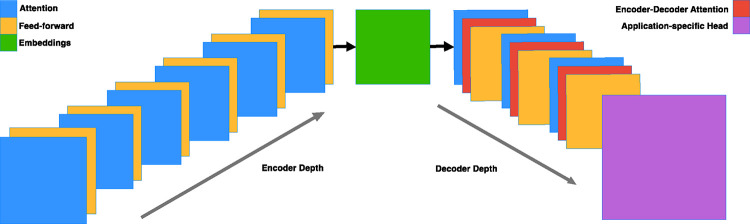
Model Architecture. The model is constructed using encoder and decoder blocks with a ratio of 2:1. Both types of blocks consist of attention and feed-forward layers, with the decoder blocks additionally incorporating the embeddings in encoder-decoder attention layers. (Sec 2.1)

ENBED is built using an encoder-decoder architecture (Fig. 1) consisting of encoder and decoder blocks, each comprised of two subcomponents: an attention layer and a feed-forward neural network. The attention layers process a sequence by replacing each element with a weighted average of the rest of the sequence, after which they are normalized and passed through the feed-forward neural network. Dropout is applied to the feed-forward network, the attention weights, and the input and output of the entire stack. The implementation is written using JAX [4] and the Flax-former library [17].

We formulate a model with 1.2B trainable parameters, with the configuration specified in Table 6. The model is encoder-heavy since idiosyncratic relationships among tokens are better encoded by devoting a larger share of parameters to these blocks. We found that adjusting the encoder-to-decoder ratio to 2:1 improved performance, with a 1% increase in Masked Language Modeling (MLM) accuracy for all model sizes over the 3:1 ratio chosen by the authors of ByT5 [33], a similar architecture built to process token-free text-to-text transformations. We also find that reducing the masked span length, which is the average number of tokens masked during pre-training, from 40 down to 20 helps in faster convergence owing to the significantly smaller vocabulary of DNA.

### Tokenization

Sequences are tokenized by breaking down the input into tokens consisting of single nucleotides. The vocabulary size is fixed at 384, with 256 ASCII characters and additional tokens added to function as <mono_space>MASK, PAD</mono_space> and <mono_space>UNKNOWN</mono_space> tokens during the training process. We require multiple <mono_space>MASK</mono_space> tokens in order to index the positions where masking has occurred and to label the targets with these indices. Although the alphabet of DNA only comprises of the four nucleic acids Adenine (A), Cytosine (C), Guanine (G), and Thymine (T), we choose to keep the whole set of extended ASCII characters since they could aid in future tasks like sequence-to-sequence transformations involving targets beyond just DNA sequences, like drug structures represented by the SMILES notation system.

This approach requires more floating-point operations (FLOPs) as compared to other tokenization methods, since it increases the tokenized sequence length for the same input DNA sequences, resulting in higher resource requirements. Although this limits us to dealing with short- to medium-length sequences, we can overcome these constraints and scale up the model by reducing the complexity of attention layers as described below.

### Attention

Attention can be understood as a soft-lookup of a query Q in a dictionary of stored keys K and values V. Attention scores are generated by calculating the similarity between Q and K, each having a dimension d, with scaled dot-product $\left(Softmax\;\left({QK}^{T}/\sqrt{d}\right)\;V\right)$ attention Softmaxbeing the most common implementation. Increasing the sequence length L can be a challenge, since this type of attention has a complexity of $O\left(L^{2}\right)$.

In order to reduce the complexity while preserving function, we modify the architecture to replace dense attention with a combination of two sub-quadratic variants of attention: (i) sliding-window attention and (ii) global attention.

#### Sliding-window attention.

Local context is crucial in analyzing DNA, since biological processes like transcription and translation work within continuous regions of a sequence. Tokens within a sliding window of radius r are used to calculate the attention scores, bringing the complexity down to O(L×r). We fix r=64 for the initial three layers and increase to r=128 in the final layers, which allows them to learn higher-level representations while having the lower layers focus on local information.

#### Global attention.

For tasks that involve classifying or annotating whole sequences, we need a mechanism that aggregates global information from the inputs, in addition to the local scores. We divide the input sequence into k blocks and calculate a global token by summing and normalizing the embeddings for every token in the block. Scores are then computed for every input token by letting it attend to the neighboring tokens (as described above) and all the global tokens, which has a total complexity of O(L(r+k)).

Hence, by choosing appropriate values for r and k relative to L, we implement a scheme to calculate attention with a sub-quadratic complexity which allows us to set an input and output length of 16384, a significant improvement over the limit of 512 tokens using dense attention with the same GPU hardware.

### Applications of Foundation Models using Transfer Learning

#### Building the foundation model.

The first step in building our foundation model is pre-training it on high-quality reference sequences. We use a procedure called Masked Language Modeling (MLM). The objective is to reconstruct tokens that have been deleted and replaced with a MASK token. This task develops the ability to understand the context and vocabulary to identify the correct elements that belong in the masked segments. Utilizing a large corpus of unlabeled data allows us to impart the model with generalizable knowledge that can be fine-tuned for specific downstream tasks. The genomic corpus is constructed by concatenating FASTA files from the NCBI sources mentioned in the Data Availability section, removing any descriptions starting with ‘>‘ and ‘N’ bases that are a result of hard-masking. We choose a masking rate of 15% over the course of pre-training. The entire corpus is supplied to a collator that handles masking, padding, and truncation to ensure that the input length is maintained. We follow a linear schedule with warmup (5% of the total training steps) using the AdamW optimizer $\left(\beta_{1}=0.9,\beta_{2}=0.99,\epsilon =10^{-6}\right)$ with a learning rate of 1e-5, a cross-entropy loss function and softmax as the activation function. We train all versions of the model with maximum input and output lengths of 16,384 tokens (base pairs). Convergence takes 120–480 GPU-hours with 8 NVIDIA A100 GPUs, determined by model size and input length.

#### Fine-tuning for downstream tasks.

We fine-tune the model by modifying the final layers into a task-specific configuration. This is called the ‘head’ of the model and is attached to the final layer of the pre-trained model. Layers are gradually unfrozen in reverse order during the course of fine-tuning, allowing the Transformer to integrate with the attached head while retaining the initial layers, thus enabling the transfer of pre-trained knowledge for downstream applications.

#### Classification head.

A fully connected (dense) layer is usually added to the output of the base model, followed by a softmax activation to produce class probabilities, typically used in sequence-level classification tasks.

#### Language modeling head.

A language modeling head comprises of a single feedforward neural network layer followed by a softmax activation function. This layer takes hidden representations from the preceding layers and outputs a probability distribution over the vocabulary. The objective is to estimate the estimate the probability of a token given the previous words in a sentence. The softmax function transforms the raw output scores into probabilities, representing the likelihood of each word or token in the vocabulary at any particular position. This process is called autoregressive generation, and we use it to perform sequence-to-sequence transformations.

### Application Domains

#### Genomic Benchmarks.

The Genomic Benchmarks (GB) dataset consists of sequences from four organisms: Human, mouse (*Mus musculus*), roundworm (*Caenorhabditis elegans*) and fruit fly (*Drosophila melanogaster*). The dataset comprises of: (i) Human enhancers from Cohn et. al. [5] and Ensembl [22], (ii) Open Chromatin Region classifications from the Ensembl build, (iii) Computationally generated data for coding and non-coding sequences (iv) Multi-class data composed of three regulatory elements (promotors, enhancers and Open Chromatin Regions), (v) Non-TATA promotor sequences imported from Umarov et. al. [31].

#### Nucleotide Transformer Benchmarks.

The Nucleotide Transformer (NT) benchmarks consist of five data sources: (i) Epigenetic marks in the yeast genome, which use experimentally obtained nucleosome occupancy values processed into positive and negative observations and to provide the following histone marks datasets: {H3, H4, H3K9ac, H3K14ac, H4ac, H3K4me1, H3K4me2, H3K4me3, H3K36me3, and H3K79me3}, (ii) A dataset [14] consisting of a mix of strong, weak and non-enhancers. (iii) Promotor sequences 300 base pairs in length around transcription start sites, divided on the basis of TATA and non-TATA box promotors. (iv) Splice site datasets composed of donor, acceptor and non-splice site sequences from phylogenetically diverse organisms.

#### Noise identification.

We generate a synthetic dataset with segments of 512 nucleotides selected at random from TeloBase [21], a comprehensive database of information about telomere motif diversity. We introduce noise based on real-world raw DNA sequencing data to generate negative samples. The process introduces insertion, deletion and base-call mismatch errors with a uniform distribution of 2% each, resulting in a simulated read accuracy of 94%. The model is fine-tuned on a sequence classification task with this labeled dataset. This process can be likened to out-of-distribution detection [12], since the negative samples would represent data that does not belong to the distribution of the training dataset.

#### Biological function annotation.

We can formulate the process of annotating genes as a sequenceto-sequence task, with the input being a DNA sequence fragment and the output containing information regarding the name, type and function of the fragment. For evaluating our model, we train it to output the biological function annotation of a given genomic input sequence up to 512 base pairs in length. We choose the following annotation types for our experiment: Coding Sequences, IncRNA, snoRNA, miscRNA, miRNA, snRNA, TEC, Processed and Unprocessed Pseudogenes. These annotations are obtained from the Ensembl dataset [22].

#### Mutation generation.

**Fig. 2.**
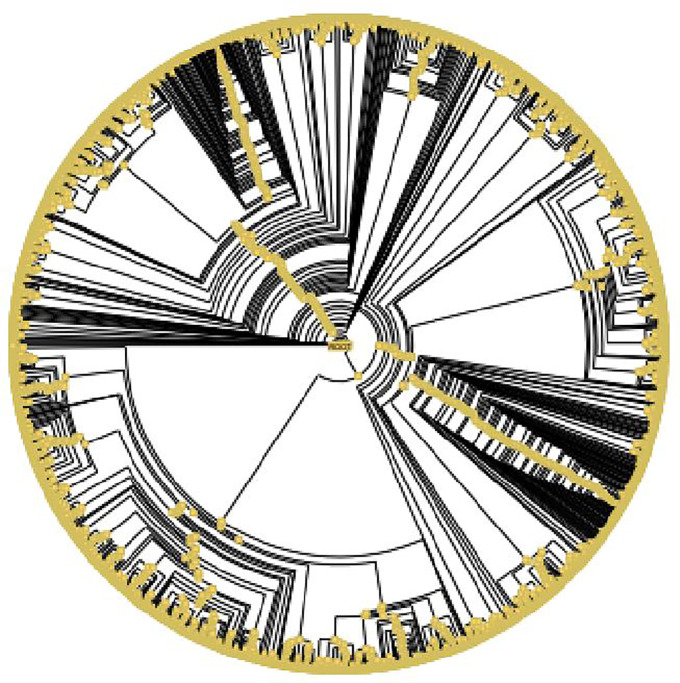
Phylogenetic Tree. Circular cladogram visualization of the Influzenza H1 gene sequences. Nodes are represented by yellow dots.

Human influenza A viruses are named based on the geographic location where the virus was isolated, the date of the isolate, and the identity of the two major surface proteins, hemagglutinin (HA) and neuraminidase (NA). We choose the HA1 sequences to create the Influenza virus mutation dataset, selecting the segments with most highly variable regions for training and validation. We obtain our source data from [3] and subset the HA1 nucleotide sequence of the H3N2 Influenza virus between 300 to 799 bp (100–266 amino-acids) to capture the Antigenic site A and B. The selected region is a part of the globular domain that occurs in a jellyroll fold of eight-stranded anti-parallel beta-sheets, containing the most commonly mutating amino-acid residues around the receptor binding site. The HA1 head also accumulates N-linked glycosylation sites over time, which are thought to mask antigenic sites from immune recognition. The glycosylation of the HA1 globular domain modulates receptor binding, stimulates host antibody responses, and shields key antigenic sites to facilitate immune evasion of the virus. By focusing on the HA1 subdomain, we aimed to evaluate the sequence-to-sequence model on a functionally important region of influenza HA that experiences significant antigenic drift and glycosylation changes. We visualize the H1 gene sequences using a circular cladogram (Fig. 2).

Candidate sequences are generated using a language modeling head with the parent sequence supplied as the input. Using a beam search $\left(N_{\text{beams}}=5\right)$, we obtain five candidate sequences which are autoregressively generated to a length of 499 bp (equal to the input). We rank the sequences using the noise identification pipeline above, and select the sequence least likely to be identified as having noise present. We identify mutations by measuring the Levenshtein distance between parent and child sequences. This metric accounts for insertion, deletion as well as in-place modifications.

## Results

**Fig. 3.**
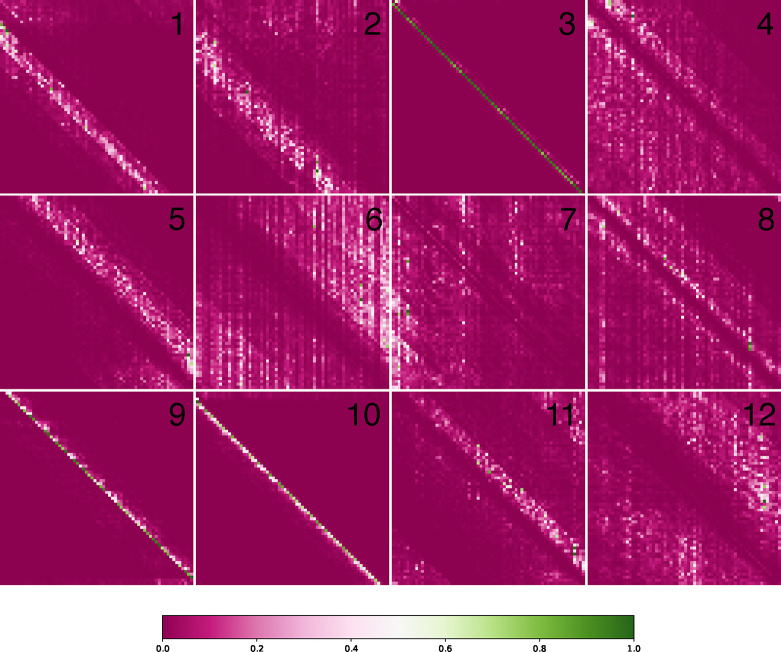
Interpreting Attention Layers. We visualize the 12 attention heads of the pre-trained ENBED (Base) foundation model. These maps are generated using the outputs from the final encoder block. The use of multiple attention heads grants the model the ability to simultaneously use a diverse range of patterns to analyze input sequences. We observe that some heads are dedicated to analyzing close neighbors (3, 9, 10) while others display a more dilated version of this phenomenon (1, 2, 5, 11). Additionally, there are heads which attempt to exclude local information and focus on a more global view of the input sequence (4, 6, 8, 12).

Upon convergence, the pre-training process yields a foundation model ready to be applied to downstream tasks. The initial layers in the pre-trained model are frozen since they contain generalizable information that helps the model build versatile internal representations of the data. We visualize these internal representations by extracting the encoder output layer and plotting attention maps in Fig. 3.

### ENBED outperforms state-of-the-art on genomic benchmark datasets

We finetune the model using a classification head (Sec. 2.4.2) using the embedding outputs from the final encoder block, on the datasets constructed by the authors of the Nucleotide Transformer (NT) benchmarks [7] and Genomic Benchmarks (GB) [15]. The results of evaluating the model on the test dataset of NT and GB are presented in Tables 1 and 2, respectively. For evaluation on the NT benchmarks, we compare our performance against the Nucleotide Transformer (v2) and HyenaDNA [7, 23], which are encoder-only and decoder-only models, respectively. For the GB datasets, we use the performance of the Convolutional Neural Network (CNN) model developed by the authors of the dataset [15] as a baseline. We also include the performance of the HyenaDNA model and the baseline Transformer developed by its authors [23].

The combination of byte-level analysis, higher quality reference sequences and improved pre-training methodology helps us exceed state-of-the-art performance in 15 out of 17 of the Nucleotide Transformer (NT) benchmarks and 6 out of 8 of the Genomic Benchmarks (GB) datasets. Byte-level tokenization helps the model internalize patterns better in the presence of variations like single nucleotide polymorphisms, and the combination of encoder and decoder blocks achieves superior performance over decoder-only methods due to its ability to focus on multiple sections of the input simultaneously and to process inputs in a context-aware manner.

### ENBED identifies noise in genomic sequences

Table 3 shows the results of the sequence-level classification on erroneous sequences using our synthetic dataset. Since competing models are trained using the GRCh38 reference assembly, they often lack information about repetitive regions due to hard-masking. We use the telomere-to-telomere assembly for pre-training the model, resulting in a signifcant performance improvement and on overall accuracy of 97.1% in the sequence-level classification task of identifying erroneous genomic data, which is significant improvement as compared to the baselines of DNABERT [19] (84.6%) and Nucleotide Transformer [7] (91.2%).

### ENBED identifies biological function annotations

ENBED is trained to identify the ten most common base-level annotations of the Human reference assembly. As shown in Table 4, we achieve an *F*_1_ score of 74.1 in this classification task, an improved score compared to contemporary models DNABERT [19] (63.2), Nucleotide Transformer [7] (67.5), and HyenaDNA [23] (72.8).

### ENBED generates mutations using sequence-to-sequence transformation

We evaluate the accuracy of ENBED in generating mutations, using an encoder-decoder Transformer with Byte-Pair Encoding (BPE) tokenization (used in previous genomic models [11]) as a baseline. We compare against BPE because this method shares similarities with byte-level tokenization by starting with the basic {A, C, T, G} alphabet, but tries to optimize the vocabulary by combining simpler words into more complex ones based on the corpus the tokenizer is trained on. While this procedure reduces the average number of tokens generated from any input sequence, it also results in reduced accuracy since modifying even a single base pair will output a significantly different tokenized sequence.

Top-1 and Top-5 Accuracy (%) scores are calculated by comparing predictions with real-world data from the Influenza Virus Resource [2], with any deviation from an exact match being classified as incorrect. Top-5 scores are calculated by selecting the best candidate from the procedure described in Sec 2.5. Additionally, we also train a version of ENBED with the encoder removed, as a comparison of the sequence-to-sequence task performance between decoder-only and encoder-decoder models.

The mean Levenshtein distance of our model predictions from real-world mutated sequences is 2.3 edits over a length of 500 bp, resulting in an average similarity of 99.5%. We can attribute the significant increase in accuracy to byte-level tokenization, since other schemes with tokens involving multiple base pairs will be unable to capture edits involving single nucleotides effectively.

### Ablation Studies

We perform ablation studies in order to examine the impact of the architectural modifications and the combination of encoder and decoder blocks.

### Encoder-decoder architecture

We study the impact of combining encoder and decoder blocks and the cross-attention links between them in Table 6. A decoder-only version of the model is constructed by stacking 24 decoder layers and is pre-trained to convergence using next-token prediction. We also construct a balanced model using stacks of 12 layers for both the encoder and decoder blocks, introducing cross-attention layers in the decoder that attend to the embeddings and the output sequence. Both models have ~ 800 M trainable parameters. We then fine-tune these models on the mutation generation task and compare with the ENBED model having a 2:1 encoder-decoder block ratio.

Introducing the encoder and cross attention leads to a significant improvement in the pre-training accuracy, demonstrating the suitability of both the architecture as well has the pre-training task, since decoder-only models are restricted to causal objectives like next-token prediction unlike encoders that can handle bi-directional information.

### Architectual modifications

We also evaluate the model’s pre-training accuracy during Masked Language Modeling using dense attention and our improved linearized attention with the same GPU memory allowance, demonstrating improved performance (Table 7) due to the greater context length.

## Conclusion

We presented the ENBED foundation model, a byte-level encoder-decoder Transformer pre-trained on an ensemble of reference genomes, with a sub-quadratic attention implementation and an input length of 16,384. We demonstrated that it generalizes well in applications involving multiple species, noisy data, and can perform a wide range of downstream tasks as summarized below:

- Achieved state-of-the-art scores in 6 out of 8 categories on the Genomic Benchmarks datasets and 15 out of 17 categories in the Nucleotide Transformer datasets.
- Demonstrated the ability to identify insertion, deletion and mismatch noise in sequences (Acc = 97.1%).
- Identified biological function annotations from sequence data with an $F_{1}$ score of 74.1
- Generated mutations in Influenza with a Top-1 and Top-5 accuracy of 76.9% and 95.4% and mean Levenshtein distance of 2.3 bp from real-world influenza mutations in 500 bp generated sequences.

## Figures and Tables

**Table 1.** Nucleotide Transformer (NT) Benchmarks.

NT Benchmark	Enformer [1]	DNABERT-2 [38]	NT (v2) [7]	HyenaDNA [23]	ENBED (no pre-training)	ENBED

H3	85.9	89.3	89.5	88.9	64.4	**90.6**
H3K14ac	63.5	75.9	76.9	80.9	51.6	**81.4**
H3K36me3	67.1	79.7	81.3	80.8	61.1	**82.7**
H3K4me1	64.6	75.8	77.7	75.8	58.4	**77.9**
H3K4me2	63.0	68.0	67.6	73.9	55.9	**75.7**
H3K4me3	56.5	67.3	69.5	77.5	50.9	**77.9**
H3K79me3	74.7	80.7	81.3	83.7	83.1	**85.4**
H3K9ac	70.8	77.1	78.0	79.3	60.2	**82.6**
H4	86.6	89.9	90.5	88.2	74.3	**91.8**
H4ac	63.8	73.1	74.9	78.4	67.2	**80.5**

Promotor (all)	95.4	97.1	97.6	96.0	94.3	**98.0**
Promotor (non-TATA)	95.5	97.2	97.6	96.0	94.4	**98.0**
Promotor (TATA)	96.0	95.5	96.6	94.1	92.9	**96.8**

Splice acceptor	91.4	97.5	**98.7**	95.8	87.8	95.8
Splice donor	90.6	96.3	**98.7**	95.8	87.7	95.4

Enhancer	72.3	75.7	77.3	75.9	65.2	**78.3**
Enhancer Types	55.4	62.0	62.6	59.5	51.4	**70.0**

We evaluate our model using the 10-fold mean accuracy (%) score of the best performing variants of the Nucleotide Transformer v2 [7] and HyenaDNA [23], highlighting the **best** and second-best scores. The scores are sourced from a leaderboard maintained by the authors of [7] on the Hugging Face platform [18].

**Table 2.** Genomic Benchmarks.

Genomic Benchmark	CNN	DNABERT	GPT	HyenaDNA [23]	ENBED (no pre-training)	ENBED

Mouse Enhancers	69.0	66.9	80.1	85.1	75.5	**90.3**
Human Enhancers (Cohn)	69.5	74.0	70.5	**74.2**	54.3	71.2
Human Enhancers (Ensembl)	68.9	85.7	83.5	89.2	83.3	**92.2**
Coding vs Intergenomic	87.6	92.5	88.8	91.3	84.2	**93.0**
Human vs Worm	93.0	96.5	95.6	96.6	90.8	**97.3**
Human Regulatory Elements	93.3	88.1	91.5	**93.8**	80.8	90.2
Human Promoter (Non-TATA)	84.6	85.6	87.7	96.6	83.4	**97.2**
Human OCR (Ensembl)	68.0	75.1	73.0	80.9	64.3	**81.9**

Accuracy (%) scores of the **best** and second-best model in the Genomic Benchmarks datasets [15]. The baseline CNN and Transformer scores was calculated by the authors of [15] and [23] respectively.

**Table 3.** Erroneous Sequence Identification.

Model	Reference	*F*_1_ Score

DNABERT	[19]	84.6
Nucleotide Transformer	[7]	91.2

**ENBED**	This paper	**97.1**

**Table 4.** Biological Function Identification.

Model	Reference	*F*_1_ Score

DNABERT	[19]	63.2
Nucleotide Transformer	[7]	67.5
HyenaDNA	[23]	72.8

**ENBED**	This paper	**74.1**

**Table 5.** Mutation Generation.

Model	Top-1 Accuracy	Top-5 Accuracy	Mean LD	Median LD

Transformer (BPE tokenization)	32.0	56.1	30.6	24
ENBED (decoder-only)	53.1	72.1	6.1	4
ENBED	**76.9**	**95.4**	2.3	1

Accuracy (%) scores of Top-1 and Top-5 candidates with the mean and median Levenshtein Distance (LD) between predicted and child sequences.

**Table 6.** Model Configurations.

Configuration	Decoder-only (no Cross-Attn.)	Base model 1:1 Enc/Dec	ENBED

Parameters	800M	800M	1.2B
*d_ff_*	3584	3584	3850
*d_kv_*	64	64	64
*d_model_*	1536	1536	1536
Encoder layers	0	12	24
Decoder layers	24	12	12
Attention heads	16	16	16

Top-1 accuracy (%)	53.1	62.0	76.9

*d_model_* denotes the size of the encoder layers, and the pooler layer, *d_kv_* is the size of the key, query, and value projections per attention head and *d_ff_* is the size of the intermediate feed-forward layer in each Transformer block. The accuracy of the top-1 candidate is evaluated using the same framework used in Table 5.

**Table 7.** MLM Accuracy.

Complexity	Input length	Accuracy(%)

Full Attention	512	68
Linearized Attention	16384	**77**

Sub-quadratic attention outperforms full *O*(*N*^2^) attention when evaluated using Masked Language Modeling (MLM) accuracy (%) with similar GPU memory usage.

## Data Availability

The Telomere-to-telomere reference sequences for Human (GCF 009914755.1) and Maize (GCA 022117705.1) and the reference sequences for *E. coli* (GCF 000008865.2), *D. melanogaster* (GCF 000001215.4), *M. musculus* (GCF 000001635.27) and *P. vivax* (GCF 000002415.2) were obtained from NCBI RefSeq [25] in FASTA format. Variant Calling Files (VCFs) for the 1000 Genomes Project [6] were obtained from the European Bioinformatics Institute. Gene annotations were obtained from GENCODE [16] and Ensembl [22]. The mutation tree was derived from the data assembled by the authors of [3], sourced from the NCBI’s Influenza Virus Resource [2]. The source code used to develop and fine-tune the foundation model has been released on Github.^1^
